# Novel Composite Electrolytes of Zr_0.92_Y_0.08_O_2-α_(8YSZ)-Low Melting Point Glass Powder for Intermediate Temperature Solid Oxide Fuel Cells

**DOI:** 10.3390/ma11071221

**Published:** 2018-07-16

**Authors:** Hongtao Wang, Ruifeng Du, Ruijuan Shi, Junlong Liu

**Affiliations:** School of Chemical and Material Engineering, Fuyang Normal College, Anhui Provincial Key Laboratory for Degradation and Monitoring of Pollution of the Environment, Fuyang 236037, China; ruifengdu1@163.com (R.D.); rjshi@fync.edu.cn (R.S.); jlliu@fync.edu.cn (J.L.)

**Keywords:** defects, electrolyte, fuel cell, ceramics, sol-gel preparation

## Abstract

In this study, Zr_0.92_Y_0.08_O_2-α_(8YSZ) powders were synthesized by the sol-gel method. The chemical physics changes and phase formation temperature of 8YSZ crystal were determined by thermogravimetry analysis and differential scanning calorimetry (TGA-DSC). 8YSZ-low melting point glass powder (8YSZ-glass) composite electrolytes with various weight ratios were prepared and calcined at different temperatures. The X-ray diffraction (XRD) patterns of the composite electrolytes were tested. The effects of synthesis temperature, weight ratio, test temperature, and oxygen partial pressure on the conductivities of 8YSZ-glass composite electrolytes, were also investigated at 400–800 °C. The result of the log*σ* ~ log(*p*O_2_) plot indicates that the 8YSZ-20% glass (700 °C) is almost a pure ionic conductor. The oxygen concentration discharge cell illustrates that the 8YSZ-20% glass (700 °C) composite electrolyte is a good oxygen ion conductor.

## 1. Introduction

ZrO_2_-based electrolytes doped, with rare earth metallic cations, are excellent oxide ionic conductors, and they are widely used in oxygen sensors and solid oxide fuel cells (SOFCs) [1,2,3,4] due to their good mechanical strength and high ionic transport numbers. During the past decades, continuous research concentrating on Y_2_O_3_-stabilized ZrO_2_ (YSZ) has been done [5,6]. For example, Caruso et al. investigated the influence of different parameters on the morphology and microstructure of YSZ powders synthesized by the sol-gel method [5].

However, SOFCs using YSZ as electrolyte membrane usually run at high temperatures (800–1000 °C). Therefore, researchers have focused on two ways to lower the operating temperature of YSZ. One strategy is to use YSZ films, and the other way is to construct composite electrolytes, which may have the combined advantages of each component [7,8,9,10,11]. A few research reports have shown that the thin film fuel cells using YSZ as membrane electrolytes generated maximum power output densities of 200–400 mW·cm^−2^ at 800 °C [12,13,14]. Singh et al. reported that the YSZ-SDC (samarium doped ceria) composite electrolyte with a weight ratio 8.5:1.5 has a higher electrical conductivity than single material YSZ at 400–700 °C [9]. It is well known that silicate, borate, mica, and other glass systems are commonly used as sealing materials in fuel cell systems [15,16,17,18]. It may be expected that using silicate low melting point glass powder, as a sintering aid, as well as composite electrolytes with improved gas tightness, durability, and better component matching, could be synthesized.

In this study, novel composite electrolytes of Zr_0.92_Y_0.08_O_2-α_(8YSZ)-low melting point glass powder were synthesized. The morphology, structure, and intermediate temperature electrochemical properties of the composite electrolytes were investigated by a variety of methods.

## 2. Experimental

We initially synthesized Zr_0.92_Y_0.08_O_2-α_(8YSZ) electrolyte via a sol-gel method using citric acid as a chelating agent as reported previously [19]. All the reagents used are analytical-grade. Firstly, Y_2_O_3_ was dissolved in nitric acid and Zr(NO_3_)_4_·5H_2_O was dispersed into distilled water. The solution was then mixed with citric acid and NH_4_OH and evaporated at 90 °C to get a gel. After gelation and ashing treatment, the obtained ash was calcined at 700 °C, 1200 °C and 1550 °C for 6 h, respectively, to get Zr_0.92_Y_0.08_O_2-α_(8YSZ) powder. The low melting point glass powder was used as a sintering aid to form composite. The composition of the low melting point glass powder is Na_2_O-CaO-SiO_2_-ZnO (Taizhou Xinhai Special Materials Factory, 300 mesh, m.p. is 550 °C). 8YSZ and low melting point glass powder were mixed with a weight ratio of four to one and heated at 700 °C, 1200 °C and 1550 °C for 2 h, correspondingly. The composites with weight ratio of 8YSZ: low melting point glass powder = 9:1 and 7:3 were also synthesized at 700 °C. These results are summarized in Table 1.

The chemical physics changes and phase formation temperature of 8YSZ crystal were determined by thermogravimetry analysis and differential scanning calorimetry (TGA-DSC) (TGA-DSC, Universal V 3.7A, TA Instruments, New Castle, DE, USA). The X–ray diffraction (XRD) (XRD, X’pert Pro MPD, Amsterdam, Netherlands)patterns of the above electrolytes were tested with a Panalytical X′Pert Pro MPD diffractometer. The morphology of the 8YSZ-20% glass (700 °C) was observed using a scanning electron microscope (SEM, S-4700, Hitachi, Tokyo, Japan) [20,21].

The conductivities vs. different synthesis temperature, test temperature, oxygen partial pressure and weight ratio in nitrogen atmosphere were tested with an electrochemical analyzer (CHI660E, Shanghai, China) at 400–800 °C. All the samples were ground into thin slices of 1.0–1.2 mm. A 20% palladium-80% silver paste with silver wires was used to fabricate the electrodes (area: 0.5 cm^2^). Oxygen concentration discharge fuel cell and H_2_/O_2_ fuel cell using the 8YSZ-20% glass (700 °C) as electrolyte were constructed [22,23].

## 3. Results and Discussion

**Figure 1:**
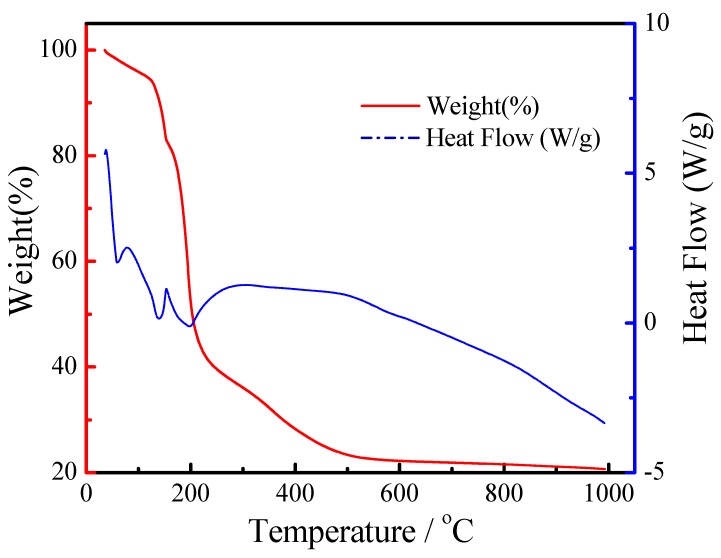
TGA-DSC test of the Zr_0.92_Y_0.08_O_2−α_(8YSZ) gel heated at 15 °C·min^−1^.

The TGA and DSC curves of the Zr_0.92_Y_0.08_O_2-α_(8YSZ) gel heated at 15 °C·min^−1^ in nitrogen atmosphere up to 1000 °C are shown in Figure 1. It can be seen that the TGA curve shows a weight loss about 7% from 35 °C to 130 °C corresponding to two weak endothermic peaks in DSC curve, which is attributed to the residual water in the 8YSZ gel [24,25]. About seventy percent of weight loss of 8YSZ gel occurred up to c.a. 500 °C. The first calcined temperature was fixed at 700 °C because there is almost no weight loss at 520 °C and above [26,27].

**Figure 2:**
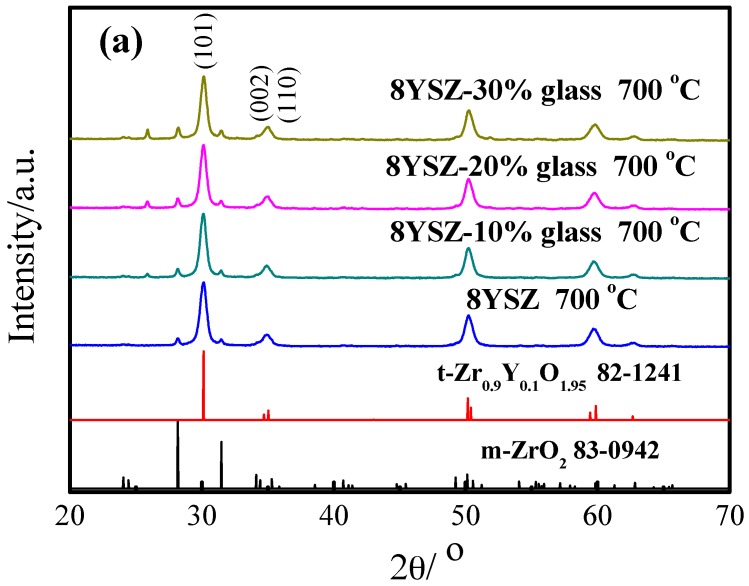

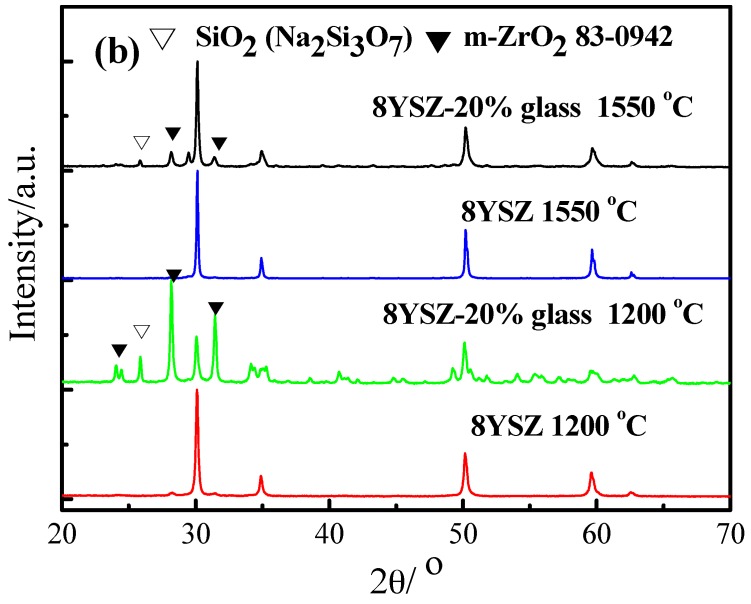
(**a**) XRD patterns of the 8YSZ, 8YSZ-10% glass, 8YSZ-20% glass and 8YSZ-30% glass calcined at 700 °C for 6 h; (**b**) XRD patterns of the 8YSZ and 8YSZ-20% glass calcined at 1200 °C and 1550 °C, respectively.

The XRD patterns of 8YSZ and 8YSZ-glass obtained with different weight ratio and calcined at different synthesis temperature are shown in Figure 2. Figure 2a shows the XRD patterns of the 8YSZ-glass with different weight ratio calcined at 700 °C, i.e., 0%, 10%, 20% and 30%. All the samples possess coexisting tetragonal and monoclinic phases, where tetragonal is the major phase. The XRD angles at 30.14°, 34.72° and 35.04° belong to the (101), (002), and (110) crystal planes of t-Zr_0.9_Y_0.1_O_1.95_ (JCPDS 82-1241), respectively. From Figure 2b, when the synthesis temperature reaches 1200 °C and 1550 °C, the XRD patterns of 8YSZ are merely tetragonal structures. However, there are still a few obvious diffraction peaks of monoclinic structure in 8YSZ-20% glass calcined at 1200 °C and 1550 °C, respectively. Mori et al. observed that the Ti^4+^-doped 8YSZ electrolyte transform from a pure cubic structure to two-phase compound containing small amount of tetragonal phase with increasing Ti content [28]. And a monoclinic-to-tetragonal phase transformation was found in 9 mol% MgO doped ZrO_2_ above 1300 °C [29]. Therefore, it is probably the high synthesis temperature and 20% weight ratio of low melting point glass leads to the appearance of monoclinic phase. Besides, a diffraction peak is observed at 2*θ* ≈ 26° may be indexed to the SiO_2_ (JCPDS 13-0026) or Na_2_Si_3_O_7_ (JCPDS 38-0019). This indicates that the Na_2_O-SiO_2_ in low melting point glass changes from amorphous to crystalline at high temperature.

**Figure 3:**
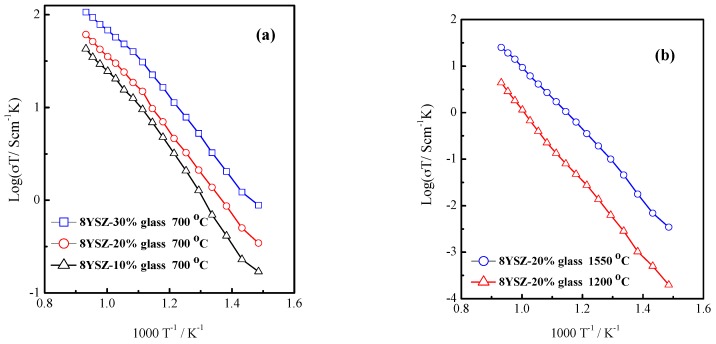
The conductivities vs. (**a**) different weight ratio of the 8YSZ-10% glass, 8YSZ-20% glass and 8YSZ-30% glass after calcined at 700 °C; (**b**) different synthesis temperature of the 8YSZ-20% glass (1200 °C, 1550 °C) in nitrogen atmosphere at 400–800 °C.

The conductivities vs. different synthesis temperature and weight ratio were tested at 400–800 °C in nitrogen atmosphere as shown in Figure 3. It is clear that the conductivities of composite electrolytes increase with the increase in glass concentration. And the highest conductivities are obtained for the 8YSZ-20% glass (700 °C), 8YSZ-20% glass (1200 °C), and 8YSZ-20% glass (1550 °C) to be 5.7 × 10^−2^ S·cm^−1^, 4.1 × 10^−3^ S·cm^−1^, and 2.3 × 10^−2^ S·cm^−1^ at 800 °C, respectively. A recent investigation by Lee et al. [29] reported that a single cubic phase of 8YSZ showed higher conductivity than 9 mol% MgO doped ZrO_2_ which has a mixed phase. Similarly, the conductivities of the 8YSZ-20% glass (700 °C) (Figure 3a) and 8YSZ-20% glass (1550 °C) (Figure 3b) are higher than that of 8YSZ-20% glass (1200 °C) (Figure 3b) which has evidently tetragonal and monoclinic biphasic structure in Figure 2b. The conductivities of the 8YSZ-20% glass (700 °C) are lower than that of 8YSZ-30% glass (700 °C) composite electrolyte as shown in Figure 3a. However, the 8YSZ-30% glass (700 °C) composite electrolyte is unstable because it will cause segregation and reduce the mechanical hardness in the molten state when the glass powder is too high in percentage.

**Figure 4:**
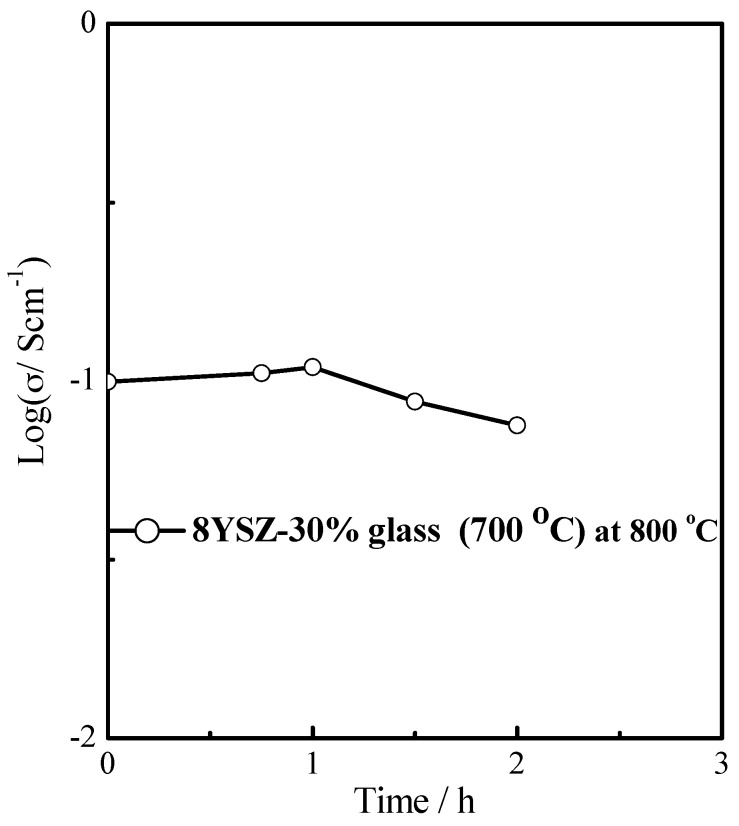
The variation of conductivity of 8YSZ-30% glass (700 °C) with time in nitrogen atmosphere at 800 °C.

Figure 4 shows the variation of conductivity of 8YSZ-30% glass (700 °C) composite electrolyte with time in nitrogen atmosphere at 800 °C. The conductivity reaches a steady state in the first hour. However, with increasing time, the conductivity of 8YSZ-30% glass (700 °C) composite electrolyte gradually decreased. This suggests that it cannot be used for long period at 800 °C.

**Figure 5:**
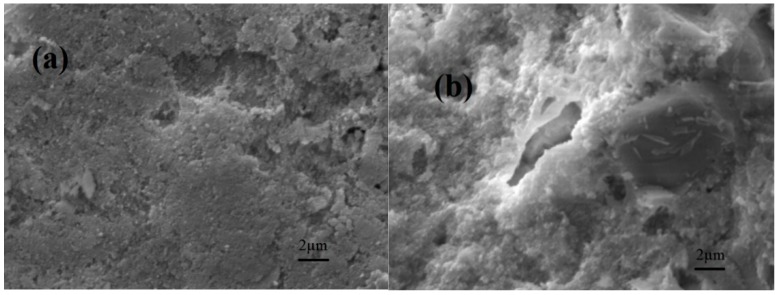
The external (**a**) and cross-sectional (**b**) surface SEM images of the 8YSZ-20% glass (700 °C) composite electrolyte.

The external (a) and cross-sectional (b) surface SEM images of the 8YSZ-20% glass (700 °C) composite electrolyte are displayed in Figure 5. The 8YSZ agglomerated with low melting point glass powder, few pores are observed and the microstructure is homogeneous after heating at 700 °C, which is attributed to high fluidity of molten glass. Figure 5 shows that the two components are evenly dispersed and intimately connected and do not react with each other due to their high chemical stability [3,5,9,11].

**Figure 6:**
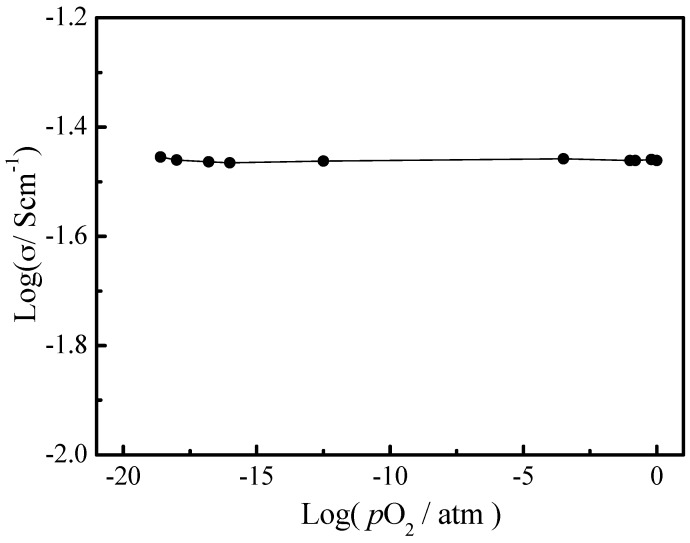
The conductivities of the 8YSZ-20% glass (700 °C) composite electrolyte as a function of *p*O_2_ at 750 °C is almost a pure ionic conductor.

In order to investigate ionic conduction of the 8YSZ-20% glass (700 °C), the relationship between the oxygen partial pressure (*p*O_2_) and conductivities was studied. As shown in Figure 6, there is almost a straight line within the whole *p*O_2_ range. The result indicates that the 8YSZ-20% glass (700 °C) is almost a pure ionic conductor [20,21,22,23]. In the *p*O_2_ range of 10^−20^~10^−15^ atm, the curve is slightly upwarped, indicating that there is a trace electron conduction in the 8YSZ-20% glass (700 °C) in reducing atmosphere.

**Figure 7:**
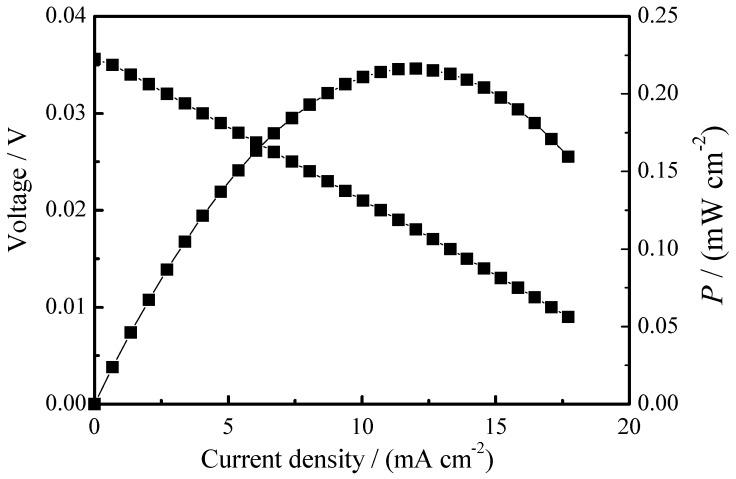
The oxygen concentration discharge cell: air, Pd-Ag|8YSZ-20% glass (700 °C)|Pd-Ag, O_2_ at 800 °C.

It is well known that ZrO_2_-based electrolyte is a good oxygen ion conductor. To study the oxide ionic conduction of the 8YSZ-20% glass (700 °C) composite electrolyte, an oxygen concentration discharge cell was tested at 800 °C as shown in Figure 7. The calculational electromotive forces (EMF_cal_) could be obtained from EMF_cal_ = $\frac{RT}{4F}$ *t*_O_ ln[*p*O_2__(A)_/*p*O_2__(B)_] when *t*_O_ = 1. The air (*p*O_2__(B)_) and pure O_2_ (*p*O_2__(A)_) are introduced into the anode and cathode, correspondingly. From Figure 7, the open circuit voltage is 35.6 mV, which is close to the calculated EMF (36.1 mV). Moreover, a stable discharge line could be seen in Figure 7. All the results illustrate that the 8YSZ-20% glass (700 °C) composite electrolyte is a good oxygen ion conductor.

**Figure 8:**
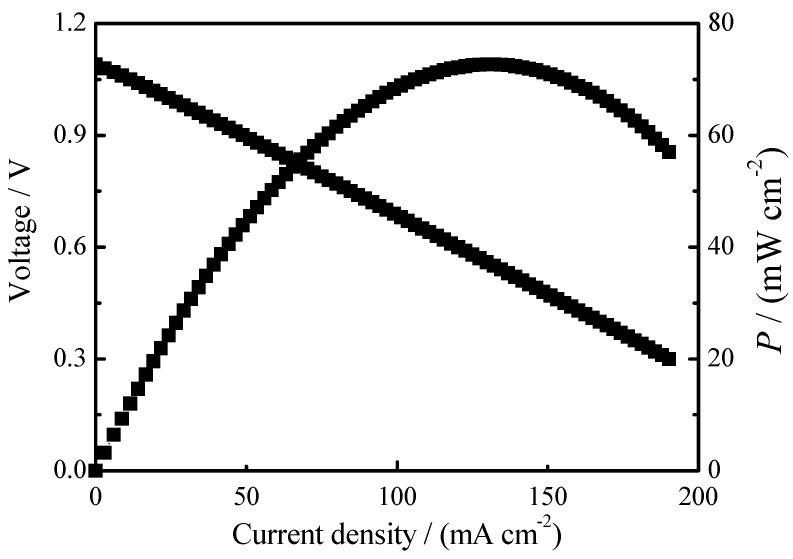
H_2_/O_2_ fuel cell of the 8YSZ-20% glass (700 °C) at 800 °C.

The H_2_/O_2_ fuel cell electrochemical performance was tested at 800 °C for the 8YSZ-20% glass (700 °C) as shown in Figure 8. It can be seen that the 8YSZ-20% glass (700 °C) reveals a high open circuit voltage (1.09 V) which means the composite electrolyte is dense [5]. The maximum power density of the 8YSZ-20% glass (700 °C) is 72.7 mW·cm^−2^ (thickness = 1.1 mm) at 800 °C. The result is lower than that previous reported cathode supported thin film fuel cell with 200–400 mW·cm^−2^ at 800–850 °C [12,13,14,30], which can be attributed to the electrolyte thickness and the electrode/electrolyte interface. Further work is in progress to optimize the composition of composite electrolytes and develop the stable and high performance solid oxide fuel cell [31,32].

## 4. Conclusions

In this study, low melting point glass powder was chosen as a sintering aid to prepare novel Zr_0.92_Y_0.08_O_2-α_(8YSZ)-low melting point glass composite electrolytes. The results of XRD indicate that the major phase in composite electrolytes is tetragonal and no diffraction peaks of low melting point glass are found. The influences of amount of additive, synthesis temperature, test temperature, and oxygen partial pressure on the electrical conductivities of the composite electrolytes were investigated at 400–800 °C. The results of the XRD and conductivities show that the 8YSZ-20% glass (700 °C) is a suitable choice. The oxygen concentration discharge cell illustrates that the 8YSZ-20% glass (700 °C) composite electrolyte is a good oxygen ion conductor. The maximum power density of the 8YSZ-20% glass (700 °C) is 72.7 mW·cm^−2^ (thickness = 1.1 mm) at 800 °C.

## Figures and Tables

**Table 1:** The samples synthesized with different synthesis temperature and weight ratio.

Sample	Synthesis Temperature	Abbreviation
Zr_0.92_Y_0.08_O_2−α_-10 wt% low melting point glass	700 °C	8YSZ-10% glass 700 °C
Zr_0.92_Y_0.08_O_2−α_-20 wt% low melting point glass	700 °C	8YSZ-20% glass 700 °C
Zr_0.92_Y_0.08_O_2−α_-30 wt% low melting point glass	700 °C	8YSZ-30% glass 700 °C
Zr_0.92_Y_0.08_O_2−α_-20 wt% low melting point glass	1200 °C	8YSZ-20% glass 1200 °C
Zr_0.92_Y_0.08_O_2−α_-20 wt% low melting point glass	1550 °C	8YSZ-20% glass 1550 °C
